# Supplementary material for: Using simulations to compare the current Davis Cup ranking system to Elo
Source: PLoS One. 2024 Feb 23;19(2):e0298188. doi: 10.1371/journal.pone.0298188 (PMC10890748; doi:10.1371/journal.pone.0298188)
Supplement: Attachment — Submitted filename: response to reviewers.docx [file pone.0298188.s001.docx]

Dear Editor

Thank you for giving me the opportunity to submit a revised version of the manuscript “Using simulations to compare the current Davis Cup ranking system to Elo”. I appreciate the time taken by you and the reviewers to provide feedback on this manuscript. I have modified the manuscript to incorporate the feedback. The changes are highlighted using track changes and relevant line numbers are included in the response to the reviewer comments below. All page numbers refer to the revised manuscript file with tracked changes.

**Reviewer 1s’ comments to the Author:**

**Reviewer Comment:** The reviewer would also like to congratulate the author for having chosen as the subject of his study a considerably relevant aspect within the discipline of tennis, namely the analysis of ranking systems.

In fact, the issue of ranking systems for both individuals and teams participating in a competition is a very topical issue in the world of tennis due to the emergence of the ITF World Tennis Number.

Thus, the reviewer considers that we are faced with a tremendously current, modern and necessary study due to its obvious implications both for the participating federations and for the players who are members of the national teams.

The author adequately justifies the importance of the topic, explains its necessity and introduces it in an appropriate manner in the opening section of the article. Possibly the only suggestion of the reviewer would be the need to include enough references to support all the claims made in the first pages of the introduction, as the first reference is not included until well into the article.

In this sense, and in general, the reviewer considers that the total number of references is relatively small, so it would be advisable to add current and appropriate references that would provide solidity to the claims made in the document.

The research design, the hypotheses proposed, the sample, the analyses used, the presentation of the results and their discussion are appropriate for an article of this quality. It seems obvious that the author has mastered the subject, deals with it correctly and presents it appropriately so that it can be understood and followed by the reader.

The tables and figures used are clear, visually attractive and provide relevant information for the understanding of the manuscript.

As per the discussion, the author compares his results with those of relevant studies and carries out a very interesting exercise of reflection on his work.

**Author Response:** Many thanks for the positive feedback on the paper and the chosen subject area. Additional introductory references to how the ATP and WTA handle protected rankings have been included (lines 118-122). An additional reference additional work modelling Elo rating has been added specifically on intrinsic strength which is a similar concept to the nation skill level.

**Reviewer Comment:** As for the conclusion, the reviewer would suggest that the author dedicate a few paragraphs to three aspects that he considers fundamental in any research work. On the one hand, reference should be made to the limitations of the study. That is, those aspects that the author identifies as obstacles to the study and the reasons for them. On the other hand, a reference to the future prospects of the research, based on the results obtained in the study. Specifically, the directions or lines of work that could be derived from the findings of this research. And finally, it would be highly advisable to dedicate a few paragraphs to the possible practical applications of the work. A kind of answer to the question "What then? In this way, the manuscript could be brought to a more appropriate conclusion.

**Author Response:** I thank the reviewer for the excellent suggestion and have added a limitations heading (line 508), a practical applications and future prospects heading with additional paragraphs (line 521).

**Reviewer 2s’ comments to the Author:**

**Reviewer Comment:** The authors want to determine whether an Elo-based approach for rating Davis Cup teams might offer an improvement over the Davis Cup ranking method. They use event simulations to evaluate the quality of ratings and rankings according to several properties they argue are desirable for a rating/ranking system. The study aim has potential interest for the implementation of the Davis Cup implementation. The authors don't provide a good case for the scientific importance of the study.

**Author Response:** I thank the reviewer for the comment. A new section, “Practical applications and future prospects” (line 521), has been added to discuss the practical applications and future prospects that may follow on from this work.

**Reviewer Comment:** The major concern I have with the study is that the Elo rating is derived at the team level with no consideration of the skill of the players who compose the team. The power of the Elo system lies in its dynamic self-correcting update, where ratings go up or down based on the latest results in a direction to make the last prediction more correct. Because the Davis Cup team competition can change drastically from round to round, this raises the question of whether a team-level Elo is an appropriate application of the tool. The goal of the Elo system is to measure an underlying skill that can't be directly observed. What is the skill that is attempting to be measured here if the composition of the Davis Cup team is changing so frequently? The author would be on firmer ground if the simulation were based on the Elo-predicted results on individual players or, perhaps, some team-level rating based on the summary of the player skills on the team at any given time.

**Author Response:** I thank the reviewer for this useful comment. It is correct to consider the option of applying the Elo rating to individual players. If the aim of the Davis Cup ranking system was to best predict tie results, this would likely be the avenue to take. However, the ranking system is required to differentiate nations for seeding and drawing ties. The text added from lines 166 to 183 includes this valuable point. This point also contributes to additional text justifying the alternative Elo optimisation on line 310. The relatively good performance of the alternative approach to defining the Elo k-factor showed that the Elo method can perform well despite this limitation.

**Reviewer Comment:** Rankings don't provide a direct way of simulating match results. The author uses skill points for each ranking to derive probabilities of team A beating team B but these are arbitrary. If other probabilities were assigned to the skill points, the results could change, which puts doubt on the validity and robustness of the findings.

**Author Response:** The reviewer makes an excellent point. However, there is insufficient data from the current competition structure of the Davis Cup to use to validate the chosen simulation win-likelihoods. When there is enough data, this point could be revisited. This limitation is stated in the “Limitations” section (line 508).

**Reviewer Comment:** Some of the criteria for a 'good' ranking system appear to be in conflict. Both 'Responsiveness' and 'Protection' could be in conflict with the skill correlation of the system, for example, if the system is too sensitive to small sample sizes in how it updates or provides a disincentive for the best players to participate.

**Author Response:** The reviewer’s comment is correct in that the different criteria require different and sometimes opposing qualities from the ranking method. The most obvious pair of criteria requiring opposing qualities are *responsiveness* vs *protection*. We want a ranking system that responds quickly enough to reflect improvement within a satisfactory fraction of a player’s career but that provides ranking protection over a year or so. Both criteria are bounding in opposite directions, but it is still possible to satisfy both.

**Reviewer Comment:** A smaller point is that the scaling factor of the Elo system should also be considered a tuning parameter as this should approximate the variation in the underlying skill distribution.

**Author Response:** As described in line 286-291, an Elo using a K-factor of k and an S parameter of s behaves identically to an Elo using a K-factor of Fk and an S parameter of Fs. The only difference is that the ratings would also be scaled by F. Therefore, one of the K-factor or the S parameter should be fixed and the other used to define the Elo. I chose to fix the S parameter at 400 and optimise the K-factor. If I had fixed the S parameter at 200, the optimised K-factors would have been half of what was found. Tuning the S parameter after optimisation would effectively be changing the K-factor after optimising its value, which would not be correct.
