# Supplementary material for: Using simulations to compare the current Davis Cup ranking system to Elo
Source: PLoS One. 2024 Feb 23;19(2):e0298188. doi: 10.1371/journal.pone.0298188 (PMC10890748; doi:10.1371/journal.pone.0298188)
Supplement: Attachment — Submitted filename: Response to reviewers 3&4.docx [file pone.0298188.s002.docx]

# Response to Reviewers

Reviewers’ comments in bold

**Reviewer #3: The authors could provide good work. However, there are some concerns to be resolved.

The abstract needs to be improved. The first sentence in the abstract, it is necessary for the authors to add a sentence to describe the problem or motivation to focus on this topic.**

I thank the reviewer for their suggestions. I assume the reviewer means “first paragraph” when they state “first sentence”. The sentence “Therefore, it is essential that the ranking method performs well with respect to required performance criteria of the International Tennis Federation.” Has been added to the end of the first paragraph of the abstract to describe the motivation for the work.

**The second sentence should provide the literature gap.**

I thank the reviewer for their suggestions. I assume the reviewer means “second paragraph” when they state “second sentence”. The sentence “Such a comparison has not previously been published.” Has been added to the 2^nd^ paragraph to highlight the literature gap.

**In the third sentence, the authors should say what you are doing, and then provide the empirical findings.**

I thank the reviewer for their suggestions. I assume the reviewer means “paragraph when they state “sentence”. The second paragraph describes what was done and the third paragraph describes the findings.

**Finally, the significance of the finding should be offered.**

I thank the reviewer for their suggestions. The final paragraph of the abstract has been changed to: “In conclusion, the current Davis Cup ranking method is performing at a standard that cannot be matched by typically optimised Elo but can be matched when an alternative optimisation method is used. Therefore, no evidence was found to suggest that the current Davis Cup ranking method could be improved upon by using Elo. However, alternative K-factor optimisation methods should be considered when applying Elo to a competition.”

**The organization of the Introduction section is very unsatisfactory, and it is very messy and hard to read. Thus, this section needs rewriting in order to make it crisp and the main points of the research methodology should be mentioned clearly. This will help the readers to appreciate the novelty of the research.**

I thank the reviewer for their suggestions. Numbered headings have been included to allow the reader to more easily follow the document which is of particular use in the introduction.

**English presentation of the paper should be modified so as to be more readable.**

I thank the reviewer for their suggestions. The English has been thoroughly checked.

**Improve the literature review. Add several pieces of research in 2019-2023. Moreover, the following references should be used:
A robust possibilistic programming framework for designing an organ transplant supply chain under uncertainty. Annals of Operations Research, 1-38.
Two-echelon electric vehicle routing problem with a developed moth-flame meta-heuristic algorithm. Operations Management Research, 1-22.
Efficient multi-objective meta-heuristic algorithms for energy-aware non-permutation flow-shop scheduling problem. Expert Systems with Applications, 213, 119077.
Designing a portfolio-based closed-loop supply chain network for dairy products with a financial approach: Accelerated Benders decomposition algorithm. Computers & Operations Research, 155, 106244.
Integration of blockchain-enabled closed-loop supply chain and robust product portfolio design. Computers & Industrial Engineering, 179, 109211.
Just-in-time scheduling in identical parallel machine sequence-dependent group scheduling problem. Journal of Industrial & Management Optimization.**

I thank the reviewer for the suggestions. The following sentence has been added:

“Simulations-based optimisation has been previously used in medical supply chain design [1]”.

It is difficult to include the remaining suggested references. The improved moth-flame optimisation used in the 2^nd^ suggested reference and the many optimisations used in the 3^rd^ suggested reference are not necessary for or relevant to the optimisation carried out in this work. Additionally, the time-saving findings are not relevant as my analysis was not time-limited. The 4^th^ suggested reference uses a Benders decomposition, not required here as we have a simple single parameter problem. I cannot see how the implementation of blockchain technology is relevant to this work with regard to the 5^th^ suggested reference and it is hard to compare the performance of nations to a portfolio of products. The optimisation methods and the speed and solution accuracy findings of the 6^th^ suggested reference are also not relevant to this work. I notice the reviewer has focussed solely on references authored by Alireza Goli. There may be better fitting additional references the reviewer has knowledge of beyond the work of Goli.

**Most of the methodological choices lack a clear motivation, and their impact on performance is not analyzed on the manuscript.**

Performance in terms of computational speed was not within the scope of this research. The optimisation described was to select the “best” k-factor for the Elo algorithm. The standard cost function for Elo is the prediction of results. this is described in section 3.2. The innovative alternative cost function is also described in section 3.2.

**On the whole, there is no clear indication of where the authors see the main innovation and value of the methodology described.**

The main methodological innovation and value is the finding that an innovative alternative optimisation for the Elo k-factor produced an algorithm that out-performed Elo optimised in the standard way, as stated in the Results, discussed in the Discussion and highlighted in the conclusion with implications stated. The main findings of the paper are not methodological – see the aim in section 2.5.

**Discuss the pros and cons of the proposed methodology in more detail.**

The limitations are covered in the Limitations section (6.2).

**The author has poorly discussed the results of the paper. One would expect to find the previous empirical work enriching the discussions of the results, but unfortunately, that has not been done.**

The reviewer is not clear as to the section they are referring to. Do they mean the results section or the discussion section? This approach has not been applied before, either to compare different ranking methods or specifically to test Elo, so there is little empirical work to compare against

**The discussion is relatively simple and insufficient. I recommend strengthening the comparison with previous research. Please compare the results in this study with those in previous studies. Discuss the study findings here. The discussion and conclusion are appropriately written and require no changes.**

This reviewer comment is quite contradictory. I will assume final sentence is what is required.

**The manuscript does not answer the following concerns: Why is it timeliness to explore such a study? What makes this study different from the previously published studies? Are there any similarly findings in line with the previously published studies? Are the findings different from prior academic studies that were conducted elsewhere, if any?**

In response to this reviewer’s comment, I will quote reviewer 1’s comments:

“The reviewer would also like to congratulate the author for having chosen as the subject of his study a considerably relevant aspect within the discipline of tennis, namely the analysis of ranking systems.

In fact, the issue of ranking systems for both individuals and teams participating in a competition is a very topical issue in the world of tennis due to the emergence of the ITF World Tennis Number.

Thus, the reviewer considers that we are faced with a tremendously current, modern and necessary study due to its obvious implications both for the participating federations and for the players who are members of the national teams.

The author adequately justifies the importance of the topic, explains its necessity and introduces it in an appropriate manner in the opening section of the article.”

**Reviewer #4: The paper presents a comparative approach to better ranking system of Davis Cup. Although, the application is new to tennis, there are no significant novel methods. In addition, the results and achievements are significant and can be implemented.
Also, the content is adequate, but it is not well-organized and the layout of the manuscript is incorrect. Please consider changing the layout of the manuscript by introducing the main points:
1. Introduction
2. Materials and methods
3. Results and discussion
4. Conclusions**

**You can also number the main and sub-headings for better tracking.**

I thank the reviewer for their comments. I have reformatted and restructured the headings to fit the proposed headings above and included numbered headings and sub-headings as suggested. The results and discussion section have been kept separate, but the sub-headings of the discussion match the relevant sub-headings in the results.
